# Regulatory T Cells in Atherosclerosis: Is Adoptive Cell Therapy Possible?

**DOI:** 10.3390/life13091931

**Published:** 2023-09-18

**Authors:** Alexey V. Churov, Yegor S. Chegodaev, Victoria A. Khotina, Vladimir P. Ofitserov, Alexander N. Orekhov

**Affiliations:** 1 Institute on Aging Research, Russian Gerontology Clinical Research Center, Pirogov Russian National Research Medical University, 129226 Moscow, Russia; 2 Institute of General Pathology and Pathophysiology, 8 Baltiiskaya Street, 125315 Moscow, Russia; 3 Moscow Aviation Institute, National Research University, 4 Volokolamskoe Shosse, 125993 Moscow, Russia

**Keywords:** regulatory T cells, FOXP3, atherosclerosis, inflammation, CAR Treg

## Abstract

**Simple Summary:**

Atherosclerosis is a vascular disease with an asymptomatic debut and development over decades. The mechanisms of progression of atherosclerosis are not completely clear. The development of atherosclerosis involves the infiltration of various types of immune cell subsets into the inner layer of vessel walls, leading to vascular inflammation. These recruited cells mainly have a pro-atherogenic effect. Regulatory T (Treg) cells maintain the balance within the immune system by regulating the activity of these pro-atherogenic immune cells, and they appear to be beneficial in controlling the initiation and progression of atherosclerosis. In particular, Treg cells control anti-inflammatory macrophage polarisation, inhibit foam cell formation, and promote pro-atherogenic Th cell responses through multiple suppressive mechanisms. Here we highlight the most recent advances in our understanding of the roles of Treg cells in the atherogenic process and discuss specific therapeutic strategies for the treatment of atherosclerosis by Treg manipulation.

**Abstract:**

Atherosclerosis is an insidious vascular disease with an asymptomatic debut and development over decades. The aetiology and pathogenesis of atherosclerosis are not completely clear. However, chronic inflammation and autoimmune reactions play a significant role in the natural course of atherosclerosis. The pathogenesis of atherosclerosis involves damage to the intima, immune cell recruitment and infiltration of cells such as monocytes/macrophages, neutrophils, and lymphocytes into the inner layer of vessel walls, and the accumulation of lipids, leading to vascular inflammation. The recruited immune cells mainly have a pro-atherogenic effect, whereas CD4^+^ regulatory T (Treg) cells are another heterogeneous group of cells with opposite functions that suppress the pathogenic immune responses. Present in low numbers in atherosclerotic plaques, Tregs serve a protective role, maintaining immune homeostasis and tolerance by suppressing pro-inflammatory immune cell subsets. Compelling experimental data suggest that various Treg cell-based approaches may be important in the treatment of atherosclerosis. Here we highlight the most recent advances in our understanding of the roles of FOXP3-expressing CD4^+^ Treg cells in the atherogenic process and discuss potential translational strategies for the treatment of atherosclerosis by Treg manipulation.

## 1. Introduction

Cardiovascular diseases are the leading cause of death worldwide. Generally, the underlying mechanism of cardiovascular pathology is atherosclerosis, which, in most cases, is the cause of stroke and ischaemic heart disease and accounts for about a quarter of global deaths [1,2]. Atherosclerosis is closely associated with impaired lipid metabolism and with the involvement of various immune cell subsets and immune cell-derived biological factors [2,3,4].

Hypercholesterolemia leads to the infiltration and accumulation of plasma low-density lipoproteins (LDLs) in the artery wall, which in turn triggers the development of local inflammation, vasoconstriction, and thrombosis. Consequently, atherosclerosis slowly progresses with the development of chronic inflammation and the formation of lipid plaques in the large and medium arteries of the body. As a rule, atherosclerosis remains asymptomatic for the first few decades of its development until rupture or erosion of the atherosclerotic plaque elicits thrombus formation that occludes the blood vessel and leads to tissue damage due to ischaemia [2,3,4].

The process of atherogenesis is complex and involves many biochemical and molecular mechanisms. Lipid retention in vessel lesions, along with some other pro-inflammatory mechanisms, elicits local inflammation with an influx of monocytes from the peripheral circulation. Further, monocytes differentiate into macrophages, which accumulate intracellular cholesterol and produce inflammatory mediators [2,3,4].

Both the adaptive and innate immune systems are involved in the pathogenesis of atherosclerosis. Macrophages are the main immune effector cell type during plaque formation in atherogenesis. However, other cell types are actively involved. CD4^+^ T cells recognise components of LDLs (such as Apo B100 protein) as antigens and are also recruited to lesion formation. Within the local environment, T cells, like macrophages, produce many mediators (mostly Th1 cytokines IFNγ, and TNFα) and contribute to the development and maintenance of inflammation. Thus, T cells are crucial in the generation and sustainment of atherosclerosis. Autoantigen-responsive T cell subsets, including Th1, regulatory T (Treg) cells, and Th17, manage plaque development [2,3,4].

Treg cells are a specialised subpopulation acting as suppressors of various immune responses [5,6,7,8]. It has been shown that Treg cells are able to inhibit immune cell functions, including T cell proliferation and cytokine production. This function makes Treg cells a promising tool for designing novel therapies for the treatment of atherosclerosis and related clinical conditions.

In this review, we describe the role of CD4^+^ Treg cells in the natural course of atherosclerosis and discuss potential opportunities for the development of Treg-focused approaches for anti-atherogenic therapy.

## 2. Biology of Treg Cells

Human Treg cells were first characterised in 2001 as a CD4^+^ T cell subset that constitutively expresses the membrane marker CD25 (IL-2 receptor alpha chain) [9,10]. In 2003, the transcription factor forkhead box P3 (Foxp3) was described as a master control gene for murine Treg cell development and function [11,12]. Subsequent studies have confirmed FOXP3 as a specific marker for human Treg cells [13].

Treg lymphocytes are highly heterogeneous and do not represent only one subset of suppressor cells. Numerous subtypes of Treg cells have been reported. However, human CD4^+^ Treg cells can be divided into two key subpopulations: natural or thymus-derived Treg cells, which differentiate in the thymus during early neonatal development, and inducible (adaptive) Treg cells, which are generated in the periphery from non-regulatory CD4^+^ T cells. These cell subsets have similar and complementary functions in the immune system but differ significantly in their development, stability, antigen-specificity, and suppressive activity [5,14]. When activated, Treg subsets may differentiate into effector Treg subsets and acquire tissue-specific functions: tissue-resident and follicular Treg cells [14,15,16].

Treg cell subsets are defined according to their surface phenotype and expression of intracellular markers (Table 1), as well as by cytokine secretion.

Transcription factor FOXP3, a member of the forkhead/winged-helix family of transcriptional repressors, is recognised as a key molecule in CD4^+^ Treg cell biology and, so far, the most specific marker used for distinguishing between Treg and non-regulatory T cells. FOXP3 controls Treg differentiation and plays a vital role in both the function and activation of FOXP3-expressing T cells [14].

Treg cells exhibit suppressor activity against a wide range of immune cells, including inhibition of proliferation and functional activity of CD4^+^ and CD8^+^ T cells, B cells, antigen-presenting cells (APCs), and natural killer cells (NK) [5,14].

Irrespective of the Treg origin, subset affiliation, and phenotype, FOXP3^+^ Treg cells exert their immunoregulatory function via direct cell-to-cell contact, changes in the metabolism of target cells, and the release of suppressive mediators. Mechanisms of Treg functional activity include ligation of inhibitory receptors (PD-1, OX40, CTLA-4), competitive local binding of IL-2 via high CD25 expression, shifting of tryptophan metabolism, as well as granzyme production and release of inhibitory cytokines: transforming growth factor-β (TGFβ), IL-10, and IL-35 [14].

## 3. Treg Numbers and Balance in Atherosclerosis and Their Atheroprotective Roles

Over the past decade, considerable attention has been paid to the idea that Treg cells control the development of inflammation and atherogenesis by restraining excessive immune activation and inducing immune tolerance in experimental models of atherosclerosis and in clinical studies.

Direct evidence indicating the relationship between Treg cells and atherogenesis was obtained in a number of animal studies. The first data on the protective role of Treg cells in atherosclerosis was obtained by Ait-Oufella et al. (2006) [30]. The authors showed that depletion of Treg cells by anti-CD25 antibodies was associated with a significant increase in atherosclerotic lesion size and elevated plaque instability in Apoe^−/−^ mice [30]. Further studies in mice have convincingly shown that defective Treg cells can contribute to the progression of atherosclerosis [31].

One of the first clinical studies on the role of Treg cells in atherogenesis was conducted by de Boer et al. in 2007 [32]. The authors examined the frequencies of Treg cells expressing FOXP3 and GITR markers in all developmental stages of human plaque formation and found that Treg cells were present at low frequencies in injured arterial intima compared to normal human vessel tissues. At the same time, high-risk lesions and unstable plaques were enriched with Treg cells compared to early atherosclerotic lesions and stable plaques, respectively [32].

Further clinical studies uncovered significant changes in the number and function of Treg cells during the natural course of atherosclerosis. To date, the data generally indicate a decrease in the number of cells and their functional activity.

For instance, several groups independently reported that the development of atherosclerosis is strongly associated with decreased numbers of Treg cells both in the peripheral circulation and in atherosclerotic lesions in patients [19,33,34,35].

In 2012, Liu et al. showed that the frequencies of Treg cells among peripheral blood mononuclear cells (PBMCs) in patients with carotid artery plaques were significantly lower than in subjects without plaques [34].

In another study by Dietel et al. (2013), it was found that the numbers of plaque-infiltrating Treg cells were associated with disease severity: the number of Treg cells decreased in patients with vulnerable plaques as compared to individuals with stable atherosclerotic plaques [35]. Interestingly, these results were inversely correlated with increased rates of mature dendritic cells (DCs) infiltrating atherosclerotic lesions [35].

Rohm and co-authors observed significantly lower numbers of Treg cells in unstable atherosclerotic lesions with elevated levels of DCs, T helper cells, cytotoxic T cells, and NK cells [19]. Thus, data in the literature indicate a decrease in the number of Treg cells, which is associated with a general imbalance of pro-inflammatory (atherogenic) and anti-inflammatory (atheroprotective) Treg cell populations and disease severity in patients suffering from atherosclerosis.

However, there is some conflicting data related to the content of Treg cells in atherosclerosis. Mailer et al. (2017) reported that hypercholesterolemia, one of the major disease risk factors for atherosclerosis, facilitates proliferative T cell responses on the systemic level and, thus, may increase the expansion of both thymic and peripherally induced Foxp3^+^ Treg cell subsets [36]. Kologrivova et al. (2019) found that in hypertensive patients, the increase in Treg cell numbers is associated with coronary atherosclerosis. The authors suggested that an increase in Treg cell numbers may constitute a compensatory mechanism that helps to counterbalance the state of chronic low-grade inflammation in these patients [37].

In addition to significant changes in Treg cell numbers, numerous studies have demonstrated that changes in the functional status of Treg cells may also contribute to atherogenesis. The natural course of atherosclerosis can lead to various Treg cell dysfunctions, such as elevated susceptibility to apoptosis [38,39], modulation of Treg cell plasticity, and migratory potential [40,41]. Impaired suppressive Treg cell activity [42] and a change in cytokine production [33,34,41] are also associated with the pathogenesis of atherosclerosis.

For example, oxidised LDLs (ox-LDLs) may promote Treg cell apoptosis, leading to a reduction in Treg frequencies [38,39]. Atherogenesis may drive Treg cell plasticity and stimulate differentiation and accumulation of Th1-like IFNγ^+^CCR5^+^ Treg cells with a unique transcriptional phenotype characterised by co-expression of Treg and Th1 lineage-specific genes. This intermediate cell subpopulation has an impaired suppressive function and may promote excessive T-cell-mediated atherogenic immune responses [41].

Liu et al. (2012) found that the plasma levels of cytokines related to Treg cells (IL-10 and TGF-β1) were lower in patients with unstable plaques than in plaque-negatives or patients with stable carotid plaques [34]. These changes were accompanied by an increase in the level of Th17 cells and the plasma levels of cytokines: IL-17, IL-6, IL-23, and TNFα. Thus, Liu et al. hypothesised that the imbalance of Th17/Treg cells and their cytokines is involved in the development and progression of atherosclerosis and may enhance plaque instability [34]. Butcher et al. (2016) showed that IFNγ, IFNα, IL-2, and IL-7 signalling pathways are involved in the regulation of Treg plasticity in atherosclerosis [41].

Compared to Treg numbers and their functional characteristics, the imbalance of Treg cells with effector immune cell subsets (such as Th1, Th2, CD4^+^CD28^null^, and Th17) appears to be a more important mechanism contributing to pro-inflammatory responses in atherosclerosis and plaque instability. For example, the Treg/Th17 imbalance is associated with atherosclerosis and acute coronary syndrome and is regulated by FOXP3/STAT5 and RORγt/STAT3 transcription factors, respectively. The Treg/Th17 ratio is modulated by enhancing Th17 proliferation or by Treg apoptosis, which are both induced by elevated levels of ox-LDLs and inflammatory DAMPs, such as HMGB1 (reviewed in [6]).

Collectively, the above data illustrates that a decrease in Treg cell numbers and their functional activity are contributors to the pathogenesis of atherosclerosis. Thus, manipulations aimed at restoring the number of Treg cells and their functions can be used in the treatment of atherosclerosis.

**Figure 1:**
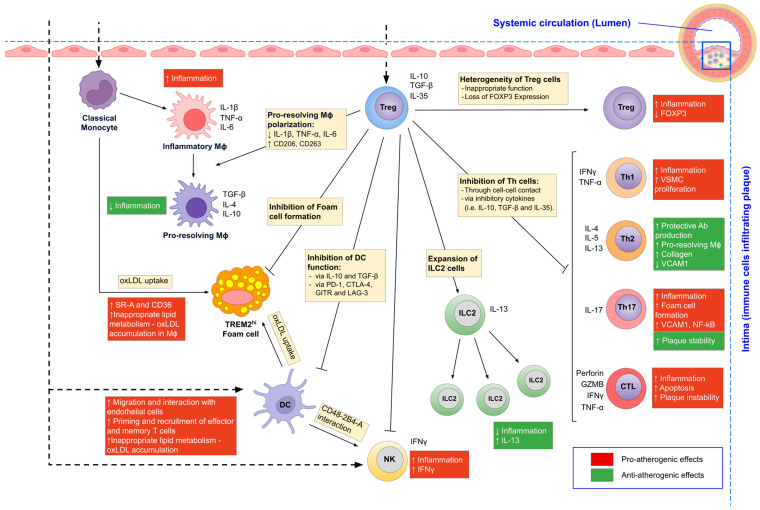
Multiple roles of Treg cells in the control of pro-atherogenic immune responses. Various immune cells, including classical monocytes, Th-cell subsets, and Treg cells, enter the systemic circulation, homing to the plaque, where APCs initiate a response by effector immune cells. Treg cells modulate immune cell responses through the production of IL-10, TGFβ, and IL-35 and through cell contact-dependent mechanisms, including Perforin and granzyme B, as well as co-inhibitory molecules such as GITR, CTLA-4, PD-1, and LAG-3. FOXP3^+^ Treg cells are heterogeneous and include cell subsets with unstable FOXP3 expression and impaired functional activity. Key pro-atherogenic effects and anti-atherogenic effects are highlighted in red and green, respectively. Mϕ, macrophages; TREM2, triggering receptor expressed on myeloid cells 2; IFNγ, interferon-γ; TNF-α, tumour necrosis factor-α; VCAM1, vascular cell adhesion molecule 1; VSMC, vascular smooth muscle cell; NF-kB, nuclear factor kappa B.

Numerous studies in recent years have provided strong evidence that Treg cells are important in regulating and restricting pro-atherogenic inflammation and may play a protective role in atherogenesis by targeting both the innate and adaptive immune responses (Figure 1).

The anti-atherosclerotic effect of Treg cells is based on a number of well-studied mechanisms, including reduction of plasma cholesterol levels, regulation of macrophage metabolism and limitation of their proinflammatory activation, functional modulation of dendritic cells, and control of atherogenic T- and B-cell responses (Table 2).

## 4. Emerging Clinical Evidence and the Rationale for Treg-Focused Therapies in Atherosclerosis

The ability of Treg cells to control a wide range of pro-atherogenic immune reactions urges us to consider these cells as a promising tool for the immunotherapy of atherosclerosis. The potential for the use of Treg cells in the treatment of immune-mediated diseases has been demonstrated in numerous clinical trials. It has been shown that Treg cell-based therapy is safe and can be quite effective without the risk of developing severe adverse effects in conditions such as transplantation or autoimmune diseases.

Studies of Treg-based therapies in autoimmune diseases began less than 10 years ago. The purpose of these clinical trials was to verify the technical challenges associated with the preparation of Treg cells and their infusions, as well as to evaluate the safety and efficiency of this approach.

The first-in-human investigation of adoptive Treg therapy for autoimmunity was conducted in 2012 as a part of the CATS1 study [52]. This phase I/IIa open-label, multicentric clinical study conducted in 4 groups of 20 patients with symptomatic refractory Crohn’s Disease has demonstrated the safety of single Treg infusions [52].

Clinical trials of Treg infusions for the therapy of type 1 diabetes mellitus (T1D) began in 2011. The first trial conducted at the Medical University of Gdansk (Poland) was a phase I randomised study of autologous Treg cells isolated ex vivo from young patients with recently diagnosed T1D [53]. In 2014, Marek-Trzonkowska and co-authors published the first favourable results from this study. This trial did not reveal any severe adverse events after infusions of autologous Treg cells and confirmed their safety [53]. In addition, 8 of the 12 study participants with T1D showed signs of remission. The safety of Treg infusions was also confirmed in another study, the results of which were published in 2015 [54]. In this trial, fourteen adult patients with T1D were distributed into four cohorts depending on the Treg dosage. The study participants received infusions of polyclonal Treg cells in amounts ranging from 0.05 × 10^8^ cells in the first cohort to 26 × 10^8^ cells for the fourth cohort. Interestingly, up to 25% of Treg cells (at their peak level in the circulation) were retained in the peripheral blood of the recipients within a year after cell infusion. According to the results of the study, no infusion-related or adverse effects were observed [54]. Other studies of the efficacy and safety of using the cells in T1D, pemphigus, and autoimmune hepatitis are currently being actively conducted (NCT03185000; NCT03239470; NCT02704338).

Despite the high potential for the use of Treg cells in the immunotherapy of atherosclerosis, to date, only a few clinical studies have been conducted using these promising cells. Moreover, these studies were aimed at evaluating Treg cells as biomarkers of the disease and not as cells for the adoptive therapy of patients with atherosclerosis.

In 2008, an observational prospective study was launched to investigate the impact of the circulating Treg cell level on the risk of developing atherosclerotic vascular damage as a complication after kidney transplantation (NCT02843867). The idea of this study was to monitor Treg cell levels in patients after kidney transplantation for a period of 5 to 10 years. The main hypothesis of the project is that a decrease in the level of Treg cells below the median is associated with an increase in the risk of atherosclerotic complications by an average of 5%. The authors of the project plan to use the results for the next study, devoted to assessing the effect of Treg expansion on the risk of post-operative atherosclerotic events after kidney transplantation.

Two more recent studies, by Johann Motsch at University Hospital Heidelberg (NCT03105427) and by Prof. Hongwei at Beijing Friendship Hospital (NCT03939338), also aimed to evaluate the role of Treg cells as a predictive marker in cardiovascular diseases.

The first study, focused on the modulation of Treg cell functions in atherosclerosis, was launched in February 2010. This randomised interventional clinical trial (NCT01183962) aimed at evaluating the effect of vitamin D on the patient’s clinical parameters and on the modulation of Treg suppressor activity in patients with atherosclerosis aged between 30 and 80 years. Vitamin D supplementation was expected to stimulate the functional activity of Treg cells. However, this study was terminated due to slow recruitment of participants and financial difficulties.

Thus, at present, only a few clinical studies of Treg cells in atherosclerosis have been carried out. Therefore, many issues remain unresolved related to the therapeutic potential of the use of Treg cells in the therapy of atherosclerosis. The first results of cell-based therapy studies obtained for autoimmune pathologies inspire some optimism.

## 5. Adoptive Transfer of Treg Cells in Atherosclerosis: Advances and Challenges

The development of atherosclerosis and the stability of atherosclerotic plaques are directly related to changes in the balance of the effector and suppressor populations of the immune system. Treg cells are key immunocytes that control immune responses and maintain tissue homeostasis. Therefore, manipulations aimed at regulating Treg cells are of interest for considering the development of personalised treatments for atherosclerosis. There are several general approaches to modulating Treg activity and Treg numbers in atherosclerosis. These approaches are summarised in Table 3.

Promising preclinical and several clinical studies have suggested that adoptive Treg transfer may be a treatment option for atherosclerosis. For therapies based on adoptive cell transfer, two kinds of Treg cells can be used: polyclonally expanded Treg cells or antigen-specific Treg cells.

In early and ongoing studies of Treg-based adoptive therapies, a general ineffective approach is used: peripheral Treg cells are sorted, polyclonally expanded ex vivo, and infused in certain quantities into the bloodstream.

However, this approach does not take into account a number of key factors. First, this approach ignores the functional state of Treg cells. Currently, Treg cells are isolated from PBMCs and expanded ex vivo. Treg cells isolated from PBMCs are heterogeneous and largely represented by cells with induced FOXP3 expression. The lack of potent and stable FOXP3 expression and steady suppressive activity are the common problems in this approach to cell therapy. Second, it is important to increase the specificity of Treg-based adoptive therapy. Antigen-specific Treg cells have been shown to be more powerful in suppressing alloimmune responses in vitro and in vivo compared to polyclonally expanded Treg cells [73]. Treg cells exert their functional activity in an antigen-specific manner: self-antigen presentation leads to Treg activation and enhances the expression of membrane-bound inhibitory molecules, which exert a suppressive effect on the target cell. The antigen-specific nature of Treg activity has been clearly demonstrated in vivo in the case of CTLA-4, which is constitutively expressed by Treg cells and T-effector cells [74]. CTLA-4 expression on activated antigen-specific Treg cells is significantly higher than on T cells. This allows Treg cells to control T cell activation by competitively blocking their access to the costimulatory molecules of the APCs. CTLA-4 expressed on Treg cells binds to CD80/86 molecules on the APC membrane and is transported into the cell through trans-endocytosis. Consequently, Treg cells regulate the APC phenotype and effectively restrain the CD28-dependent activation of naive T cells by limiting their access to costimulatory ligands [74].

Thereby, infusions of polyclonally expanded Treg cells with unknown antigen specificity cannot effectively inhibit the target cells and suppress undesirable immune responses and can lead to unwanted side effects such as systemic immune suppression and reactivation of latent infections. Thus, the use of polyclonal infusions in clinical trials is often the main reason for the low efficacy of adoptive therapy.

Recently, new highly effective therapeutic approaches based on adoptive therapy with genetically engineered Treg cells have emerged, which can overcome the barriers to the use of Treg cells for immunotherapy of atherosclerosis and other immune-inflammatory diseases.

These approaches include the application of cells with genetically modified TCR [69,70] or with the expression of highly specific chimeric antigen receptors (CARs), as well as the use of genome editing techniques such as CRISPR/Cas9 (clustered, regularly interspaced, short palindromic repeats/CRISPR-associated protein 9) [75,76,77,78].

CAR technology is a very promising tool, allowing T cells to be reprogrammed to overcome the limitations of native T cells. CAR-modified T cells have already been successfully applied for the treatment of certain types of cancer. Therefore, this technology can also be effective in the case of Treg cells.

As opposed to polyclonally expanded lymphocytes, CAR-modified Tregs specifically recognize antigens by binding directly to the protein of the target cell membrane. This process does not require peptide presentation by MHC II molecules. Thereby, CAR-Tregs can significantly increase the efficiency of cell therapy [71].

However, obtaining CAR-Treg cells with persistent suppressive activity is a technologically challenging process. Similarly to CAR-expressing T cells, infusions with CAR Treg cells may induce the so-called cytokine storm and neurotoxicity [79]. This is a very serious obstacle to the use of Treg cells for clinical purposes. In addition, the identification and selection of targets for targeted therapy and for CAR engineering also involves difficulties, especially in diseases with an autoimmune component, such as atherosclerosis.

Another approach to developing effective adoptive Treg therapy in atherosclerosis is to create modified Treg cells capable of homing to sites of inflammation. In a study by Bonacina et al. (2019), Treg cells from LDLr knockout mice were retrovirally transfected with chemokine receptors, including CXCR1 [72]. As a result, CX3CR1-expressing Treg cells showed selective homing towards the atherosclerotic plaque with high expression of the CXCR1 ligand, CX3CL1. Moreover, the authors also reported that CX3CR1^+^ Treg cells contribute to a decrease in the level of inflammation and an increase in atherosclerotic plaque stability [72].

As we have previously mentioned, one of the key issues for the clinical use of Treg cells is ensuring stable FOXP3 expression to stabilise and enhance the functional activity of Treg cells. Another important issue is maintaining Treg viability ex vivo, which also directly depends on the stability of FOXP3 gene expression. CRISPR/Cas9 genome editing technology or its advanced counterparts can offer an appropriate solution. The application of the CRISPR/Cas9 technique allows for rapid site-specific genomic targeting [80]. In the case of Treg cells, the CRISPR/Cas9 system is of considerable interest as a tool for epigenetic DNA modification at the FOXP3 locus as well as at other DNA sites important for Treg suppressive activity. An experiment with murine primary T cells has shown that a modification of a baseline gene editing technology with a mutant Cas9 protein lacking endonuclease activity (dead Cas9; CRISPR/dCas9) can induce the required epigenetic changes contributing to stable Foxp3 expression [81].

Thus, the feasibility of Treg anti-atherosclerotic therapy and the relative safety of this approach have been demonstrated in a few pilot clinical trials. Further research should focus on how immunosuppressive therapies can be combined with Treg infusions. The use of Treg cells in combination with other therapies tailored to an individual patient holds the greatest promise for the future.

In this regard, the role of new diagnostic approaches that allow choosing the optimal treatment options is increasing.

Recent progress in omics-based technologies such as CyTOF, CITE-seq, and scRNA-Seq offers new opportunities for high-resolution immune cell profiling in atherosclerotic lesions [82,83,84,85]. For example, Wang et al. (2022) analysed the immune cell landscape of atherosclerotic plaques using scRNA-Seq. The authors identified a set of pro- and anti-atherogenic immune cell types, including regulatory T cells, as well as subtypes of macrophages and foam cells [82]. ScRNA-Seq offers unique advantages over bulk RNA sequencing or flow cytometry, allowing the analysis of cell plasticity, intercellular communication at the site of inflammation, and other important data.

The aforementioned omics-based approaches can be used as state-of-the-art methods for profiling immune cell alterations in heterogeneous atherosclerotic plaques, allowing better patient stratification and a more personalised approach to atherosclerosis management.

## 6. Conclusions

A number of studies have shown that CD4^+^ Treg cells are crucial in the maintenance of peripheral tolerance and have an important role in the control of atherosclerosis-related inflammation. Therefore, Treg cells are a promising target of major research efforts focused on immune-modulating therapies against atherosclerosis. Developing anti-atherosclerotic Treg-based therapies faces several challenges related to the biosafety and functional activity of Treg cells. However, rapid progress in genetic, epigenetic, and molecular aspects of cellular immunology gives hope for a fast-track solution.

## Figures and Tables

**Table 1:** Some phenotypic markers of human Treg cells.

Marker	Description and Function	References
CD25^high^	IL-2 receptor α-chain, which is involved in Treg cell activation and proliferation.	[5]
FOXP3	Transcription factor (forkhead box P3), expressed by all Treg cells and regulates their activation and/or differentiation for the development and function.	[17]
CD127^low^	IL-7 receptor α-chain, is down-regulated on Treg cells, also correlates with FOXP3 and the suppressive function of human CD4^+^ Treg cells.	[18]
CD39	The co-expression of CD39 and CD73 ectoenzymes induces the hydrolysis of ATP to produce adenosine, which has a suppressive effect on T-cells.	[5]
CD45RO	Memory T cell-associated marker (isoforms of the leukocyte common antigen), plays an important role in TCR signal transduction.	[5]
HLA-DR	Major histocompatibility antigen (MHC) class II. Up-regulated through activation of Treg cells.	[5,19]
CTLA-4	Cytotoxic T-lymphocyte antigen-4 (CTLA-4), acts as a potent negative regulator of immune responses and controls the suppressor activity of Treg cells. Modulates T-regulatory, T-follicular regulatory and T-follicular helper cells to control B-cells responses.	[3,5]
LAG-3	Lymphocyte-activation gene 3, is involved in the cell-contact dependent mechanism of Treg-mediated suppressive activity.	[14]
GITR	Glucocorticoid induced TNFR family-related gene, has a high surface expression on activated Treg cells and low quantities on naive and memory T cells. Inhibits the immunosuppressive activity of Treg cells.	[5]
CD62L	L-selectin, a cell adhesion molecule, decelerates lymphocytes and is involved in the homing of T cells to secondary lymphoid organs.	[5]
TGF-β1	Transforming growth factor β. Pleiotropic immunoregulatory cytokine, regulates the functional activity of Treg cells.	[20,21]
GARP	Glycoprotein A Repetitions Predominant (GARP), also known as Leucine Rich Repeat Containing 32 (LRRC32). GARP is related to the bioavailability and activation of TGF-β and mediates upregulation of Foxp3.	[22,23]
Helios	The zinc finger transcription factor, mediator in T lymphocyte immune homeostasis and a marker of T cell immune tolerance, which regulates the expression of IL-2 in Treg cells.	[24,25]
PD-1 (CD279)	Programmed cell death-1, regulates the balance between Treg cell activation/tolerance/exhaustion, and also controls antigen-specific T cell responses.	[26]
TIGIT	TIGIT is a T cell immunoreceptor with Ig and ITIM domains, which is highly expressed on Treg cells and inhibits T cell activation and proliferation.	[27,28]
Basigin/ Emmprin (CD147)	Is involved in T cell activation and proliferation, plays a key role in the cytotoxicity to human neurons, as well as negatively regulates T cell responses by selective inhibition of specific downstream elements of the Vav1/Rac1 route.	[29]

**Table 2:** The mechanisms of the protective action of Treg cells in atherosclerosis.

Function	Treg-Mediated Mechanism	Effect
Regulation of macrophage cholesterol metabolism	Treg cells downregulate the expression of scavenger receptor class A (SR-A) and CD36 preventing the accumulation of lipids in macrophages [43,44].	Inhibition of foam cell formation
Regulation of macrophage polarisation toward M2 phenotype	Treg cells may favor the differentiation of pro-inflammatory M1 macrophages to M2 macrophages by releasing IL-10 [43,44].	Reduction in inflammation and pro-inflammatory cytokine production
	Treg cells may increase the stability of plaques by inducing M2-macrophages mediated collagen synthesis and vascular smooth muscle cell proliferation [45].	Improving the stability of atherosclerotic plaques.
Regulation of pro-inflammatory T cell subsets	Treg cells suppress Th1- and Th17-mediated immune responses by various direct or indirect inhibitory mechanisms, including secretion of cytokines [34,44,46,47].	Reduction of activation, proliferation, and induction of apoptosis of pro-atherogenic T cells.
Regulation of pro-inflammatory and regulatory B cell subsets *	Treg cells may attenuate follicular B2 cell responses by diminishing their maturation, survival and by inhibiting antibody production [48].	Reduction of antibody production. Decreased pro-inflammatory cytokine activity.
	Follicular Treg cells may activate B regulatory cells and facilitate their suppressive function [48,49].	Diminishing the differentiation of pro-inflammatory CD4^+^ cells into atherogenic follicular T cells.
Regulation of APCs	Treg cells modulate APCs maturation and function by cytokines (IL-10 and TGF-β) and by surface molecules (PDL-1/2, CTLA-4, LAG-3) [50,51].	Inhibition of APCs co-stimulatory potential and subsequent reduction in activation of effector cells.

* This is a potential mechanism. To date, there is too little data on the role of Treg subsets in the regulation of B-cells in atherosclerosis.

**Table 3:** Potential therapeutic strategies for Treg manipulation in atherosclerosis.

Approach	Examples	Therapeutic Effect
Modulation of Treg function by drugs [45,55,56,57,58,59]	Mycophenolate mofetil Rapamycin Fingolimod Pioglitazone Statins	Increase in Treg numbers; Treg/T effector ratio restoration
Diet and nutrients [60,61,62]	Low cholesterol diet Vitamin D3 supplementation Vitamin B17 supplementation	Increase in Treg numbers; Treg/T effector ratio restoration
Antibodies and cytokines to control Treg cells [63,64,65]	IL-2 CD3 antibody Granulocyte colony-stimulating factor (G-CSF)	Treg expansion and increased Treg-associated cytokine production
Treg-inducing vaccines [66,67]	HSP60/65 and LDL-based Treg-inducing vaccines	Reduction in atherosclerotic lesions and induction of Treg cell numbers in the spleen and lymph nodes
Adoptive therapy with conventional Treg cells *	Infusions of ex vivo expanded polyclonally activated Treg cells [53,54]	Increase in circulating polyclonal Treg cells with unknown antigen specificity
	Infusions of in vitro stimulated antigen-specific Treg cells * [68]	Increase in circulating antigen-specific Treg cells
Adoptive therapy with engineered Treg cells [69,70,71,72]	TCR-engineered Treg cells * CAR-modified Treg cells * Plaque-homing Treg cells	An increase in the number of antigen-specific cells or cells with desired characteristics.

* Based on studies on other diseases.

## Data Availability

Data sharing not applicable.

## Abbreviations

APCs	antigen-presenting cell
CAR	chimeric antigen receptor
CAR-T cells	chimeric antigen receptor T cells
Cas9	CRISPR-associated protein 9
CITE-seq	cellular indexing of transcriptomes and epitopes
CRISPR	clustered, regularly interspaced, short palindromic repeats
CTLA-4	cytotoxic T-lymphocyte antigen-4
CyTOF	cytometry by Time-Of-Flight
DAMPs	damage-associated molecular patterns
DCs	dendritic cells
FOXP3	transcription factor forkhead box P3
GITR	glucocorticoid induced TNFR family-related gene
G-CSF	granulocyte colony-stimulating factor
HMGB1	high-mobility group protein B1
HSP60/65	heat shock 60 and 65kD proteins
IL	interleukin
IL2	interleukin-2
IFNγ	interferon-γ
LDLs	low-density lipoproteins
LAG-3	lymphocyte-activation gene 3
NKs	natural killer cells
PBMCs	peripheral blood mononuclear cells
PD-1	programmed cell death-1
PDL-1/2	programmed cell death 1 ligand 1 and 2
RORγt	retinoic acid-related orphan receptor γt
STAT	signal transducer and activator of transcription
scRNA-Seq	Single-cell RNA sequencing
TCR-T cells	T cell receptor-engineered T cells
TGF-β	transforming growth factor β
Th	T helper cell
Th1	T helper 1
Th17	T helper 17
TIGIT	T cell immunoreceptor with Ig and ITIM domains
TNFα	tumor necrosis factor-α
Treg	regulatory T cell
T1D	type 1 diabetes mellitus
